# The Influence of Marriages of Consanguinity on the Offspring

**Published:** 1862-06

**Authors:** E. H. Neyman

**Affiliations:** Student of Med. Depart. of Lind University


### ARTICLE XVII.

EXTRACTS FROM AN INAUGRAL THESIS ON

### THE INFLUENCE OF MARRIAGES OF CONSANGUINITY ON THE OFFSPRING.

By E. H. NEYMAN, Student of Med. Depart. of Lind University.

I have chosen a curious subject, yet one that is of paramount importance not only to the physician but to the whole human family; a subject that deserves the pen of a grand lama in the medical Thibet, instead of mine. Yet, while I may not be able to clothe it in a magnificent array of language, or dive very deep down into the fountains of logic, I shall endeavor to compile, as briefly as possible, facts relating to the influence of marriages of consanguinity on the offspring.

Every one is familiar with the frequently observed fact, that when any sort of people are so confined as to compel them to intermarry for any length of time, those who are nearly their blood relations, that as an inevitable result such people weaken in mind and body.

Veterinary surgeons of eminence have always studiously held up to horse raisers, the physiological fact, that horses originally of the first stock, would dwindle down to mere dwarfs when interbred for a long period. Yet, while scientific men have been so careful to note these facts, when they relate to our domestic animals—and consequently to our pockets, scientific medical men have, with either unpardonable forgetfulness, unfortunate ignorance, or culpable thoughtlessness, failed altogether to point out the vital importance of the observance of the same law in the human animal. Even if they have deigned to give it a passing glance, it was merely a superficial one, not sufficiently accurate, precise, or extended to recommend it to the mass, whom it is calculated to benefit. Since, however, the paper of Dr. BEMIS, before the American Medical Association for 1858, has excited so much physiological and social interest, there has been a new era inaugurated in the investigations of this medico-domestic subject.

A gentleman of my acquaintance, possessing a very fine dog, and being desirous to continue in the possession of the blood, bred to a very fine bitch, which dying directly after the littering of her pups, it became necessary, in order to propagate the blood, to breed one of this litter to the dog. This was carried on for several generations, each successive generation being manifestly inferior to the one just preceding it. Of the last litter there were seven; five of which died with convulsions. A still more curious fact is, that the mother of this litter died of the rabies. Without stopping to discuss the intricate question of whether this disease necessarily only results from the introduction of a specific virus, or if it may not be generated *de novo* in the system of the canine, I will merely state that a highly respectable minority advocate the latter opinion, which, if true, I must ask that this be considered a cause of spontaneous rabies.

After this somewhat lengthy, although I hope not altogether useless degression, I would call your attention to the consideration of the following statistical facts:—The wise and benevolent Dr. HOWE, in his report of the idiocy of Massachusetts, found seventeen families wherein the parents were blood relations. Of these families there were ninety-five children; of whom one was deaf, one blind, one a dwarf, twelve scrofulous, and *forty-four idiots.* Of the eight hundred and seventy-nine such families in Ohio, there were three thousand nine hundred children, *twenty-four hundred* of whom were intellectually or physically *deformed.*

The venerable FELIX ROBERTSON, in the *Nashville* (*Tenn.*) *Medical and Surgical Journal*, No. 3, Vol. XVIII, reports an instance of the marriage of *second cousins*, whose families had been in the habit of intermarrying. As the product of this marriage there were eleven children; three of whom were physically deformed, although the parents were perfectly symmetrical. Here we have an aggregate of eight hundred and ninety-five families, consisting of four thousand children, of whom *two thousand four hundred and seventy* were either mentally or physically deformed. By a reference, we see that the number of children are not decreased, but that *three-fifths are deformed.* It is true that some of the parents were unhealthy and others intemperate; but to rebut against this is the fact, that the evil effects, like that of phthisis pulmonalis, sometimes lies dormant or inert in the first generation, to be visited with unmitigated severity in some succeeding one. But we need not dive into the uncertainties and confusing labyrinths of statistics to find evidences of its ravages. Every practitioner, let his experience be ever so limited in scope of observation or inconsiderable in extent, must have frequently met with instances, without any other ascribed cause. I myself am acquainted with several such instances, which not only possess the merit of being entirely new, but also of being marked in their application.

One family whose females have never, within the scope of the "oldest inhabitant," changed their names, but with some egotistical impression that their family name was superior to any other, have with scrupulous exactness chosen their "other halves" from their blood relations; while the members of the "stouter sex," of the same family, have with equal exactness and scrupulous faithfulness, chosen their "helpmates" by the same rule, save and except one, who by mistep of nature has been cheaten out of his mate, as he seems to be an odd one: and now, woe to hymen, lives the life of single-blessedness.

This family are not only effeminate in mind and body, but some are subject to epileptic convulsion, a disease which I believe follows close on the heels of such violations. I am also acquainted with another family, of whom there are four idiots, three of the lowest grade, not even possessing the ordinary intelligence of the brute creation, while they possess all its passions in a magnified degree. About three years ago, when first commencing the study of medicine, I attended my preceptor on a visit to see a child with convulsions. Its mother, a very intelligent woman, informed us that it was her third child; that it had been idiotic ever since its birth; that it did not possess the instinct common with the lower animals; that it manifested no knowledge of its surroundings; had never walked, although three years old. It died from convulsions. And from the marked resemblance between the parents, I was subsequently induced to make inquiries, and ascertained that its parents were *cousins.*

Now, here are three instances. Of the first, I do not know of any such close intermarriages that do not develope such results. Whether the *mental deficiency* is a consequence or a cause of the marriages is more than I am prepared to answer. If it is a cause, however, it would be difficult to reconcile the physical deformities. Of the second instance, candor demands that I state that the father while, an intelligent enough man was an inveterate drinker, a perfect specimen of chronic satisnes. How far this influence might have went to produce the results it is impossible to determine. That it contributed a part cannot be denied, possibly all. While in the third instance, I know of no influence that would modify or restrict its full application. The parents were intelligent, not inebriate or physically deformed.

But to the consideration of the subject soberly. Should not the physician—the guardian of mankind, address himself to the reason of men, show them the magnitude of this sin; hold up the evil unstript of its horrid deformities, that they may see and appreciate the enormity of the crime, and seeing it may avoid it. A healthy offspring should be *the first* consideration. No parent is so inhuman as to mar or wish to mar the intellectual or physical symmetry of his children. Yet many do do it—ignorantly do it. Any step towards the solution of this problem should be hailed with joy; and he who points out the true causes of deformity in our progress is entitled to the gratitude of every enlightened man and woman. If then consanguinity in marriage is a cause—it should be placarded at every corner until it becomes as familiar to the people as "household words;" and then, if their moral sense did not teach them to find a remedy in the avoidance of the cause, actual legislative interference should be resorted to. If it was not discountenanced socially it should be legally.

